# Essential Oils from Fruit and Vegetables, Aromatic Herbs, and Spices: Composition, Antioxidant, and Antimicrobial Activities

**DOI:** 10.3390/biology10111091

**Published:** 2021-10-25

**Authors:** Soumi De-Montijo-Prieto, María del Carmen Razola-Díaz, Ana María Gómez-Caravaca, Eduardo Jesús Guerra-Hernandez, María Jiménez-Valera, Belén Garcia-Villanova, Alfonso Ruiz-Bravo, Vito Verardo

**Affiliations:** 1 Department of Microbiology, Campus of Cartuja, University of Granada, 18071 Granada, Spain; soumidemontijop@ugr.es (S.D.-M.-P.); mjvalera@ugr.es (M.J.-V.); aruizbr@ugr.es (A.R.-B.); 2 Department of Nutrition and Food Science, Campus of Cartuja, University of Granada, 18071 Granada, Spain; carmenrazola@correo.ugr.es (M.d.C.R.-D.); ejguerra@ugr.es (E.J.G.-H.); belenv@ugr.es (B.G.-V.); vitoverardo@ugr.es (V.V.); 3 Institute of Nutrition and Food Technology ‘José Mataix’, Biomedical Research Center, University of Granada, Avda del Conocimiento sn., Armilla, 18100 Granada, Spain; 4 Department of Analytical Chemistry, Faculty of Sciences, University of Granada, Avd. Fuentenueva s/n., 18071 Granada, Spain

**Keywords:** essential oils, DPPH, ABTS, food spoilage, antiradical activity

## Abstract

**Simple Summary:**

The use of essential oils (EOs) in the food industry is a popular research topic, as they have antioxidant and antimicrobial activity and could be used as ingredients directly in food or as bioactive component in food coating and food packaging. Thus, the study of their antioxidant and antimicrobial activity is a crucial step to evaluate their use in food packaging/coating. In this work, we evaluate the antioxidant and antimicrobial activities of 13 EOs from herbs, spices, fruits, and vegetables. Briefly, the EOs from aromatic herbs and spices showed the highest antioxidant and antimicrobial activity. Fennel essential oil reported the lowest antioxidant activity, however it showed very good antimicrobial activity against *Botrytis cinerea*, one of the post-harvest pathogen microorganisms in fruits and vegetables.

**Abstract:**

In the field of food preservation, encapsulated Essential Oils (EOs) could be the best non-toxic and eco-friendly tool for food preservative applications substituting the chemicals ones that have several disadvantages for the environment and health. Thirteen commercial EOs from plants, fruits, and vegetables were characterized by GC-MS. The antioxidant activity was measured by DPPH and ABTS techniques. Antimicrobial activity was assessed by agar well-diffusion method and the Minimum Inhibitory Concentration (MIC) by agar dilution method against six bacteria, *Candida albicans*, and *Botrytis cinerea*. All the EOs tested have demonstrated antioxidant activity in the range of IC_50_ 0.01–105.32 mg/mL. Between them, cinnamon EOs were the best, followed by oregano and thyme EOs. Fennel EO showed the lowest radical scavenging. MIC values ranged from 0.14 to 9 mg/mL. *C. cassia*, thyme, and oregano EOs were the most effective against the bacterial species tested, and the yeast *C. albicans*. On the contrary, citric fruit EOs showed low or no inhibition against most bacterial strains. The percentages of inhibition of mycelia growth of *B. cinerea* ranged from 3.4 to 98.5%. Thyme, oregano, mint, and fennel EOs showed the highest inhibition.

## 1. Introduction

Food security is supported in four main pillars: food access, food utilization, food stability, and food preservation. The latter mainly consists of the degradation and microbial contamination that can affect food. Food spoilage is one of the problems that should be avoided. A lot of chemical preservatives have been developed and proved to have a significant contribution in controlling this degradation. However, they have often raised negative concerns to the consumers as they are not from a green source. They need long term degradation cycles, they are environmental toxicology and have potential risks of carcinogenesis and teratogenesis in humans and animals. Due to this, essential oils (EOs) and their active components are under study for their potential use as preservatives due to their wide antibacterial, antifungal, antimycotoxigenic spectrum, and antioxidant properties [1]. Therefore, the use of EOs in food industry is growing, as they could be directly added to edible products or used for active packaging and edible coatings [2]. Thus, the use of essential oil in food industry has a double action due to their antioxidant and antimicrobial properties [3]. Moreover, the bioactive compounds contained in the EOs could also be used for pharmaceutical and cosmetic applications [4].

Overall, EOs are products that can be isolated from leaves, bark, seeds, fruit peels, roots, flowers, buds, and stems, namely, from agro-industrial by-products in the majority of cases. The antioxidant activity of the EOs is related to the complex mixture of terpenes, terpenoids, and phenylpropanoids that compose them. Among others, it was noticed that carvacrol, thymol, and eugenol are able to inhibit the oxidation processes [5].

The same happens with the antimicrobial activity of the EOs, but in this case the mechanism depends on the specific chemical components. The most common mechanism seems to be related with the alteration in the membranes, modifying their dynamicity and permeability, and in consequence releasing the cytoplasmic constituents. However, the effect is different for each microorganism depending on the variability of the membrane thickness, composition, and cellular metabolic activities [1].

The limitations that could carry the use of EOs as preservatives in food can be due to the intense aroma, high reactivity, hydrophobicity, reduced solubility, and possible negative interaction with the matrices, leading to changes in the intestinal absorbance and organoleptic properties. However, nowadays they are avoidable due to the new mechanisms studied and developed for encapsulating those EOs. These methods can improve the stability and solubility of the EOs, protecting them from the environmental interactions [6].

Therefore, for agriculture and food fields, encapsulated EOs could be the best non-toxic and eco-friendly option for preservative applications as active food packaging, enhancing food shelf life. In this context, one of the goals of the SHEALTHY project is to find essential oils that could be incorporated in food packaging in order to obtain active packaging/coating for fruit and vegetables. In this way, it is important to identify essential oils with antibacterial and anti-fungus properties that can act against foodborne pathogens, e.g., *Botrytis cinerea*, a necrotrophic fungus that affects several fruits, causing the production of high quantities of fruit wastes. Thus, the aim of this work is to evaluate and compare the antioxidant and antimicrobial activities of 13 commercial EOs from plants and fruit and vegetables. To achieve this, the EOs were characterized by GC-MS. The antioxidant activity was evaluated by DPPH (2,2-diphenyl-1-picrylhydrazyl) and ABTS (2,2′-Azino-bis (3-ethylbenzothiazoline-6-sulfonic acid) diammonium salt) assays. The antimicrobial activity was tested against microorganisms that usually spoil food and are dangerous for human health.

## 2. Materials and Methods

### 2.1. Reagents and Samples

All reagents were purchased from Sigma-Aldrich (St. Louis, MO, USA). Solvents were from Honeywell (Wabash, Lafayette, IN, USA) except ethanol, which was from PanReac (Barcelona, Spain). Water was purified using a Milli-Q system (Millipore, Bedford, MA, USA).

Thirteen essential oils were purchased from a local supplier; seven of them hail from aromatic plants and spices, and others were from fruits and vegetables (Table 1). All samples were obtained by hydrodistillation (purity 100%). Selection of the essential oils took the literature data and the availability in the Spanish market into account.

### 2.2. DPPH Free Radical-Scavenging Capacity

DPPH radical scavenging activity was assayed with a method proposed by several authors [7,8]. Briefly, 2.9 mL of 100 µM DPPH (in methanol or *n*-hexane depending on the polarity of the essential oil) was mixed with 100 μL of each essential oil at different concentrations. They were incubated during 30 min at 25 °C and were measured at 517 nm. The antioxidant activity of the EOs was expressed as the concentration of extract that inhibited the DPPH radical formation by 50% (IC_50_). IC_50_ for each sample was calculated by elaborating a curve where is represented the concentration (mg/mL) and the percentage of inhibition calculated as in the following Equation (1):

Inhibition (%) = (blank − sample)/blank × 100(1)

where “blank” is the absorbance of DPPH with the sample replaced with methanol and “sample” is referred to the absorbance of the DPPH mixed with the essential oil or the control. Ascorbic acid was used as a reference of positive control from 0.0001 to 0.5 mg/mL.

### 2.3. ABTS Acid Cation Radical-Scavenging Capacity

This technique was developed by Re at al. [9] in which the monocation ABTS^•+^ is generated by oxidation of the ABTS with potassium persulfate in the dark at room temperature for 12–24 h. For the analyses, 2 mL of 7 mM ABTS solution was added to 20 µL of different concentrations of each sample, and they were measured at 734 nm after 30 min of incubation at 30 °C. The antioxidant activity of the EOs was expressed as the concentration of extract that inhibited the ABTS radical formation by 50% (IC_50_). The IC_50_ for each sample was calculated by elaborating a curve where is represented the concentration (mg/mL) and the percentage of inhibition calculated as in the Equation (1). Ascorbic acid was used as a reference of positive control from 0.0001 to 0.5 mg/mL.

### 2.4. Antimicrobial Activity

#### 2.4.1. Test Microorganisms

The antimicrobial activity of essential oils was tested against *Staphylococcus aureus* (*S. aureus*), methicillin-resistant *Staphylococcus aureus* (MRSA), *Escherichia coli* (*E. coli*), *Salmonella enterica* serovar Typhimurium (*S. Typhimurium*), *Listeria monocytogenes* (*L. monocytogenes*), *Candida albicans* (*C. albicans*), and *Botrytis cinerea* (*B. cinerea*). All bacterial strains and *C. albicans* were isolated from various clinical samples in the Microbiology Service of the Virgen de las Nieves Hospital (Granada, Spain) and were stored as glycerol stocks and reactivated by incubation in tryptic soy agar at 37 °C for 24 h. *B. cinerea* was obtained from the Spanish Type Culture Collection (CECT 2100) and was maintained and grown at 25 °C in Sabouraud dextrose agar.

#### 2.4.2. Agar-Well-Diffusion Method

For bacteria and *C. albicans*, antimicrobial activity was assessed following the method described by Hayes and Markovic [10] with modifications, as follows: 15 mL of molten Mueller Hinton agar were poured into sterile Petri dishes and allowed to set to form a base layer. An 8 mm diameter stainless steel cylinder was placed over the base layer, and 10 mL of molten Mueller Hinton agar containing the inoculum were poured over the surface of the base layer and left to set. For the preparation of inoculate, cultures of the strains were suspended in buffered saline solution until reaching a turbidity corresponding to 0.5 McFarland standard and were inoculated in molten Mueller Hinton agar to obtain a final concentration of approximately 1 × 10^6^ CFU/ mL. After solidification of the upper layer, the cylinders were carefully removed and 25 μL of essential oils were added into the resulting holes. As a positive control for antimicrobial activity, ciprofloxacin was used at 2 mg/mL for MRSA, 0.1 mg/mL for *L. monocytogenes*, and 0.01 mg/mL for the remaining bacteria, and ketoconazole at 0.01 mg/mL for *C. albicans*. After incubation at 4 °C for 30 min to allow extracts to diffuse into the medium, the plates were incubated at 37 °C for 24 h. The inhibition zone diameters were measured (mm) and recorded as the mean ± standard deviation (SD). Three replicates were conducted.

For *B. cinerea*, Sabouraud dextrose plates were prepared following the previous procedure but testing one extract per plate. After 30 min incubation to allow the extract to diffuse, a 4-mm plug of mycelium was placed at a 3 cm distance from the essential oil. The plates were incubated for 7 days at 25 °C. The growth control consisted of Sabouraud dextrose plates inoculated with mycelium alone. Ketoconazole at 20 µg/mL was used as antimicrobial control. The percentage of mycelium inhibition for each essential oil was calculated by measuring the area of fungal growth and comparing it to the control using the ImageJ software [11]. Three replicates were conducted.

#### 2.4.3. Determination of Minimum Inhibitory Concentration (MIC)

MIC values were assessed by agar-dilution method as follows: from 288 mg/mL solutions in 98% ethanol of each essential oil, serial dilutions in sterile water supplemented with 0.5% Tween 80 were prepared (144–0.56 mg/mL). A total of 5 mL of the essential oil’s dilutions were added in 15 mL of molten Mueller Hinton or Sabouraud agar, which was prepared previously with three-quarters of its volume of water, and plated onto sterile Petri dishes. The essential oils were tested in concentrations ranging from 72 to 0.14 mg/mL for bacteria and *C. albicans*, and from 72 to 0.07 mg/mL for *B. cinerea*. Bacterial suspensions corresponding to 0.5 McFarland’s standard were adjusted to obtain a concentration of approximately 1 × 10^6^ CFU/mL, and a spot of 10 μL of each bacterial suspension was added onto the plates. *B. cinerea* was inoculated placing a 4-mm plug of mycelium at the center of each plate. A plate containing 5 mL of sterile water supplemented with 0.5% Tween 80 in 15 mL of molten Mueller Hinton or Sabouraud agar prepared and inoculated as before, was used as a growth control. All experiments were performed in duplicate. The MIC was defined as the lowest concentration of the essential oils that completely inhibited microbial growth after incubation at 37 °C for 24 h for bacteria and *C. albicans*, and 25 °C for 7 days for *B. cinerea*.

### 2.5. Determination of Essential Oil Compounds by GC-MS

Essential oils were diluted in trichloromethane and analysed by GC-MS according to Ben Lajnef et al. [12]. Separation was achieved using an Agilent 7890A GC coupled to a Waters QUATTRO micro^TM^ GC mass spectrometer. The compounds were separated on a capillary column DB-5MS (30 m × 0.25 mm; f.t. 0.25 μm) purchased from Agilent Technologies (J&W Scientific, Folsom, CA, USA). Oven temperature was set at 40 °C for 2 min, after that the temperature increased from 40 to 250 °C at 3 °C min^−1^, and remained at 250 °C for 10 min.

MS detector parameters were set at: scan range: 40–450 *m*/*z*; solvent delay time: 3.0 min; transfer line temperature 250 °C; ion source 230 °C; and ionisation energy 70 eV. Carrier gas was (He) at a flow of 1.0 mL min^−1^. Samples were injected in splitless mode. GS-MS chromatograms are showed in Supplementary Figure S1.

### 2.6. Statistical Analysis

Pearson’s correlations between antioxidant methods were evaluated using Statistica 6.0 (2001, StatSoft, Tulsa, OK, USA).

## 3. Results and Discussion

### 3.1. Composition of the Essential Oils

Essential oils were analyzed by GC-MS and a total of 56 compounds were detected. Table 2 reported the relative amounts (%) of each compound determined in aromatic plant EOs.

According to the literature [13], cinnamon samples presented different composition according to the part of the plant that was used for the extraction. Briefly, eugenol was the main compound in *C. zeylanicum* that was extracted from leaves; on the other hand, cinnamaldehyde was the first compound in *C. cassia* sample that was extracted from bark.

Oregano EO showed carvacrol as the main compound (84.5%) followed by *p*-cymene, γ-terpinene and thymol. Similar composition has been reported by Diniz do Nascimento et al. [4]; as remarked by the same authors, the oregano essential oil composition is highly influenced by the part of the plant and the agronomic and technological processes.

*Thymus* EO composition is also affected from the different parts of plant that are used for extraction (among agronomical and processing factors). The samples that were analyzed in this work proceed from a mix of flowers and leaves; thymol was the first compound accounting for more than 60%, followed by *p*-cymene, γ-terpinene, carvacrol, and linalool, respectively, accounting for about the 90% of total compound. The same compounds were described by Diniz do Nascimento et al. [4] in thymus EOs.

β-myrcene was the first compound in *Rosmarinus* EO, the second one was camphor, followed by eucalyptol and α-pinene; the present composition is in the same order of magnitude than that reported by Diniz do Nascimento et al. [4].

Mentha EO reported the typical compounds previously found in this matrix [4] and are characteristic of this EO. Briefly, menthol and menthone accounted for 69.5% of the total compounds, followed by menthyl-acetate, eucalyptol, isomenthone, isomenthol, and mentho-furan. Other minor compounds that are usually found in this plant are piperitone and neomenthol acetate.

Finally, according to Porres-Martínez et al. [14], 1,8-cineole (eucalyptol) followed by camphor, camphene, and β- and α-pinene were the main compounds of sage EO.

Table 3 reports the composition of essential oil obtained from fruits and vegetables.

*Apium* EO showed the limonene as main compound accounting more than 70% followed by β-eudesmene; similar composition was noticed by Zorga et al. [15]. Allyl phenoxyacetate was identified as a third compound; it was recently found in celery leaves EO by Stan et al. [16].

As expected, citrus samples reported limonene as the main compound. Its content ranged from 87 to 99%. The lowest content was detected in citrus lemon sample; all the other ones showed a content higher than 99%. β-pinene and γ-terpinene were the second and third compounds, respectively, in citrus lemon EO (6%). Similar results were reported by Singh et al. [17]. All *Citrus* samples, except orange essential oil, did not comply with the ISO standards; this could be justified with the provenience of the raw material (Turkey) as reported by Singh et al. [17].

*F. vulgare* EO reported high amount of anethol that was about 97% of total compounds. Other compounds were limonene and α-pinene; fenchone and estragole were also detected in small amounts. These results agreed with those reported by Ferioli and co-workers [18] in sweet fennel.

### 3.2. Antioxidant Activity of the Essential Oils

The antioxidant activity of the essential oils was evaluated by two different assays such as DPPH and ABTS. Table 4 shows the results of DPPH assay.

**Aromatic plants EOs.** Cinnamon EOs have demonstrated to have the strongest antioxidant activity between the others and the minor differences with ascorbic acid. From them, *C. zeylanicum* leaf EO is slightly better with an IC_50_ five times lower than *C. cassia* bark EO.

Comparing with other authors, this *C. zeylanicum* leaf EO has higher free radical-scavenging than the EOs from *Cinnamomum* leaves as *C. tamala* (IC_50_: 1.65 mg/mL) [19], *C. griffithii* (IC_50_: 0.082 mg/mL), *C. macrocarpum* (IC_50_: 0.099 mg/mL) [20], and *C. malabathrum* (IC_50_: 1.7 mg/mL) [21]. These differences are due to their content in eugenol, as they only reached EOs with a content between 38.5–52% [19,20], while ours had 95.2% (Table 4). The same happens with the EO reported by Srirmavaratharajan et al. [22] from *C. wightii* leaves that contain 72.6–85.9% of benzyl benzoate as major compound, causing them to have an IC_50_ of 3.49 mg/mL, much higher than the reported in this work. Moreover, it has 23 times higher antioxidant activity than other commercial EO from the same *Cinnamomum* specie leaves (IC_50_: 0.23 mg/mL) that had 48.8% of eugenol [23]. Therefore, the antioxidant activity of *Cinamomum* leaf EO can be attributed to the content in eugenol.

*C. cassia* bark EO has reported similar IC_50_ to other authors in other varieties of *Cinnamomum* bark EOs, such as *C. altissium* (IC_50_: 0.04 mg/mL) [24] and *C. griffithii* (IC_50_: 0.07 mg/mL) [20], and lower than others (IC_50_: 0.10 mg/mL) [20,23], although in all cases the major compounds are different. It is remarkable that they did not name the compound cinnamaldehyde, which is the major compound reported by several studies [25], and it is in concordance with this work in which it has been found in an amount of 91.9%.

Following them, *O. vulgare* flower/leaf and *T. vulgaris* EOs have also demonstrated high antioxidant activity compared with the others, occupying the third and fourth positions, respectively. The IC_50_ obtained for the *O. vulgare* EO with major compound carvacrol (84.5%) is in concordance with other authors in EOs obtained by hydrodistillation as Hamada et al. [26] with an IC_50_: 0.30 mg/mL (48.4% of carvacrol), and Sokmen et al. [27] who reported IC_50_: 0.31 mg/mL (64.3% of carvacrol). Boskovic et al. [28] also agreed with us, reporting an IC_50_ of 0.33 mg/mL for the *O. vulgare* EO obtained from a Serbian company.

*T. vulgaris* EO has demonstrated slightly lower antioxidant activity than those obtained by hydrodistillation (IC_50_: 0.159–0.243 mg/mL) [29,30] maybe as the differences in composition. They reported thymol as major compound at concentrations of 36.5–55.3% followed by carvacrol 28.7–29.8% and *p*-cymene 10–11.2%. In this case, we have found thymol 61.2%, *p*-cymene 16.7%, and carvacrol 2.6%, so the difference can be attributed to the reduced content in carvacrol compared with them. However, it is in the range of values reported by Boskovic et al. [28] (IC_50_: 0.48 mg/mL) and Aazza et al. [31] (IC_50_: 0.26 mg/mL) in commercial ones from Serbia and Morocco, respectively.

After those four, the EOs with higher antioxidant activity are from *R. officinalis* leaf and *M. piperita* leaf. *R. officinalis* leaf EO has demonstrated less effectivity than those obtained by other authors that have reported IC_50_ from 0.52 to 3.48 mg/mL [32,33,34,35,36]. However, in contrast, the scavenging activity is better than the reported by Risaliti et al. [37] who obtained 25% of inhibition with 4.23 mg/mL of *R. officinalis* EO from a Greek company (our EO would need 3.77 mg/mL for this 25% of inhibition). They found lower content in β-myrcene (0.9% in front of 30.75%), camphor (11.7% in front of 20.7%), and β-pinene (8.3% in front of 11.9%), despite the fact that its content in eucalyptol is higher (48.7% in front of 14.8%). This seems to indicate that the minor compounds also contribute to the reducing power.

The antioxidant activity shown by *M. piperita* EO is in concordance with that reported by Wu et al. [38] (IC_50_: 22.77 mg/mL) in an USA commercial sample, and higher than that reported by Fatemi et al. [39] (IC_50_: 25.80 mg/mL) in an EO obtained by hydro-distillation, and Stanojevic et al. [40] (IC_50_: 58.41 mg/mL) in a EO from a Serbian company. All of them had very similar composition being the major compounds menthol (38.4–52.4%), menthone (13.8–24.9%), menthyl acetate (3.9–6.5%), and eucalyptol (3.9–5.6%), which is in concordance with us (menthol 39.7%, menthone 29.8%, menthyl acetate 6.1%, and eucalyptol 4.9%).

Following them is *S. lavandulifolia* EO. It has demonstrated higher IC_50_ than other *S. lavandulifolia* leaves EO obtained by hydro-distillation (IC_50_: 0.97–8.31 mg/mL) [41,42]. This lower antioxidant activity can be attributed to the inversion in amount in the major compounds, namely camphor (20.3–33.6%) > eucalyptol (15.0–22.2%) > α-thujene (14.9–21.4%) in front of eucalyptol > camphor > camphene that are found in our *S. lavandulifolia* EO. Risaliti et al. [37] who used *Salvia triloba* leaves EO from a Greek company achieved a 25% of inhibition with 4.47 mg/mL and ours could achieve that with 14.5 mg/mL, although the compositions are very similar (*S. triloba* eucalyptol (46.68%) > camphor (10.5%) > α-pinene (8.5%) > camphene (6.7%) > β-pinene (6.7%), and *S. lavandulifolia* eucalyptol (38.6%) > camphor (23.6%) > camphene (7.7%) > α-pinene (5.5%) > β-pinene (5.1%)). However, it seems to have better antioxidant activity than other EOs from other varieties, such as *Salvia kiangsiensis* of which, according with Fang et al. [43], 10 mg/mL are needed to have 4.3% of inhibition, meanwhile with ours only 1.5 mg/mL would be necessary, and the compositions are totally different.

**Fruit and vegetables EOs.***A. graveolens* seed EO is the vegetable EO that has shown higher antioxidant activity. However, its activity is lower than that reported by Hassanen et al. [44] who, with 0.9 mg/mL of *A. graveolens* seed EO hydro-distillated, achieved 74.3% of inhibition. With this concentration of our EO, we could reach only 35% of inhibition. Although the compositions are similar, they obtained an EO with higher amount of β-selinene (27.0% in front of 9.8%) which could be the responsible of this increment in the antioxidant activity.

Taking into account the citrus EOs, the order according with their antioxidant strength is *C. reticulata* > *C. sinensis* > *C. paradisii*. They have very similar composition with D-limonene as major compound counting for 99% in all cases. However, the difference in the reducing power between them could be due to its content in the second major compound β-myrcene that counts for 0.40, 0.36, and 0.31%, respectively. Comparing with Kamal et al. [45], they obtained 24.1, 18.5, and 14.0% of inhibition with 0.1 mg/mL of *C. reticulata, C. sinensis*, and *C. paradisii* EOs, respectively, which corroborate the order and differences between them found in this study. The values of IC_50_ obtained for *C. sinensis* and *C. paradisii* EOs are in the range of the values reported by Phi et al. [46] (28.5–63.43 and 45.7–86.3 mg/mL, respectively). If comparing our *C. sinensis* peel EO with *C. sinensis* leaves EO, the studies revealed that those that come from leaves have much higher antioxidant activities with IC_50_ between 0.75–1.49 mg/mL [47]. This is mainly attributed to the composition that consists of 16.9% β-pinene, 13.8% D-limonene, and 7.5% of β-ocimene as major compounds. In this group of essential oils, it can be appreciated that the antioxidant activity can clearly be attributed to the limonene content, but also to the content in other monoterpenoids. For the *C. paradisii* peel EO, although the composition reported by Kaanin-Boudraa et al. [48] and Ou et al. [49] are very similar to that reported here, they obtained an IC_50_ of 40 mg/mL, amounting to half of our total. Another *Citrus* EO, *C. limon* peel EO, has been evaluated apart from the rest due to its different composition found (Table 3). Compared with the others, it has lower D-limonene (87.0%), β-pinene (6.0%), and γ-terpineno. Ben Miri et al. [50] found that *C. sinensis* EO had the double antioxidant activity than *C. limon*, while in this study, both EOs have demonstrated similar IC_50_. However, the antioxidant activity found is in concordance with Guo et al. [51], who reported 32.8% of inhibition with 30 mg/mL. Moreover, it has been demonstrated that *C. limon* peel EO has lower antioxidant activity than *C. limon* leaves EO according with Fancello et al. [52] who reported an IC_50_ of 11.9 mg/mL.

The EO which has shown least antioxidant activity between all tested is *F. vulgare* var. dulce EO. The result obtained is very far from those reported by Kalleli et al. [53] from Tunisian (IC_50_: 0.20–0.49 mg/mL) and French (IC_50_: 0.59–0.63 mg/mL) *F. vulgare* seeds EOs. However, it is closer to the values reported by Ahmed et al. [54] for Chinese samples (IC_50_: 15.66 mg/mL) and totally in concordance with the Egyptian ones (IC_50_: 141.82 mg/mL). According to them, the Tunisian and Chinese ones agree with us about the major compound, anethole (54.3–78.3%) but they revealed higher amounts of estragole (17.1–20.2%), L-fenchone (7.4–12.1%), and D-limonene (2.4–4.7%). Otherwise, the French one had as major compound estragole (44.7–88.9%), with lower amounts of anethole (14.0–36.3%). These compositional differences could be responsible of the changes in the antioxidant activity.

**Figure 1:**
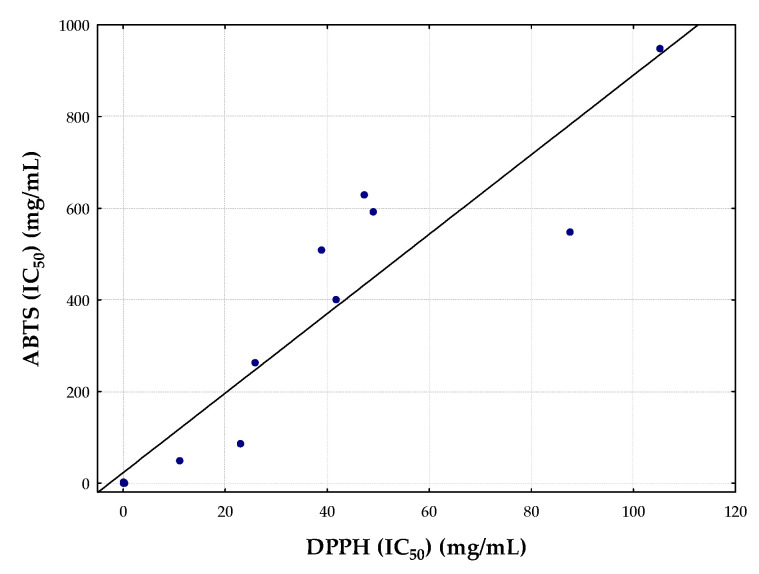
Correlation between the IC_50_values obtained by DPPH and ABTS assays in aromatic plants and fruits and vegetables EOs.

**Correlation DPPH vs. ABTS.** All the exposed data obtained by the DPPH technique is in concordance with the results obtained with the ABTS technique. As shown in Figure 1, there was significant correlation between them (r = 0.9291, r^2^ = 0.8681, and *p* < 0.001), which indicated that both techniques could be used to investigate the antioxidant activity of plants and fruit and vegetables EOs although they have not been found enough references to compare.

### 3.3. Antimicrobial Activity against Bacteria and C. albicans

Of 13 essential oils tested, 11 showed inhibitory activity against one or more bacteria or *C. albicans* (Table 5). Those essential oils that showed diameters of the zones of inhibition higher than or equal to 28 mm (8 mm well-diameter included) were considered to have a strong inhibitory effect, between 16 and 28 mm as moderately active inhibitors, between 12 and 16 mm as mild inhibitors, and less than 12 mm as no or low inhibitors [55]. Thus, Eos obtained from *C. cassia, T. vulgaris*, and *O. vulgare* proved to be strong inhibitors against most of the bacterial species tested, both Gram-negative and Gram-positive, and the yeast *C. albicans.*

Cassia bark essential oil was the most effective oil against all the strains tested showing zones of inhibition that ranged between 28.0 and 55.7 mm of diameters and higher than inhibition zones of the antimicrobial agents. Previous research is consistent with our results as essential oils of *C. cassia* have shown potent antimicrobial activity against *L. monocytogenes*, *E.coli*, *S. Typhimurium* [56], *Candida glabrata*, and *C. albicans* [57]. Firmino et al. [58] showed that essential oils extracted from cassia bark, as well as its main component, *trans*-cinnamaldehyde, in a concentration range of 0.25 to 0.50 mg/mL, inhibited the growth of the planktonic forms of *S. aureus* and *E. coli*, and reduced biomass in biofilms of both bacteria by more than 99.9%. Trans-Cinnamaldehyde is an unsaturated aldehyde that possesses an acrolein group (α,β- unsaturated carbonyl moiety) which is essential for antimicrobial activity [59]. Trans-Cinnamaldehyde has been shown to possess substantial antimicrobial activity against Gram-positive and Gram-negative bacteria, including *L. monocytogenes*, *S. aureus*, *S. Typhimurium*, *E. coli*, and *Pseudomonas aeruginosa* [56,60,61,62]. At sublethal concentrations, this compound is capable of inhibiting cell division by acting on the FtsZ protein, but at higher concentrations, it has a bactericidal action as it affects the integrity of bacterial membranes [63]. The antimicrobial activity of the essential oils of cassia observed in this work is attributable to trans-cinnamaldehyde, which represented 91.9% of the extract.

Of particular interest is the strong antibacterial activity of the essential oils of thyme and oregano EOs, herbs used frequently in gastronomy, against all the bacterial strains tested, as most of them have been implicated as causal agents of foodborne disease outbreaks and food quality degradation [64,65]. This strong antimicrobial activity is in concordance with other studies. Silva et al. [66] reported that thyme and oregano EOs showed significant antibacterial activity against *L. monocytogenes*, *S. Typhimurium*, *S. aureus*, and *E. coli*, and Bozin et al. [67] found a strong antibacterial activity of oregano and thyme EOs, even on multiresistant strains of *E. coli*, *S. Typhimurium*, and *S. aureus*. Our results showed inhibition diameters similar or higher than those of the ciprofloxacin against the tested bacterial strains, and higher than those of ketoconazole diameters against *C. albicans*.

The efficacy of these EOs can be attributed to the activity of phenolic compounds carvacrol and thymol, the major compounds of oregano and thyme essential oils, respectively. In this work, oregano EO was mainly composed of carvacrol (84.52%) and thymol (1.62%), whereas thyme oil contained 61.21% of thymol and 2.58% of carvacrol. Carvacrol has been reported to be active against *C. albicans*, *L. monocytogenes*, *E. coli*, *S. Typhimurium*, *S. aureus*, *Shigella sonnei*, and *Shigella flexneri* [56,68,69,70,71] and thymol has shown activity against *E. coli*, *S. Typhimurium*, *S. aureus, L. monocytogenes, S. sonnei, S. flexneri*, and *Bacillus cereus* [56,70,71,72]. Thymol is structurally analogous to carvacrol, but have a free hydroxyl group, the radical essential for antimicrobial activity [69], at a different location on the phenolic ring. Both compounds interact with the cell membrane, making it permeable due to the introduction of lipophilic group into the ordered structure of the lipid bilayer [63,73].

The essential oil obtained from *C. zeylanicum* leaf showed moderate inhibition against bacterial strains with inhibition zones between 14.3 and 21.7 mm, and strong inhibition for *C. albicans*. Regarding antimicrobial agents, this essential oil showed inhibition zones similar to ciprofloxacin against bacterial strains, but higher than those of ketoconazole against *C. albicans*. In agreement with our results, Ebani et al. [74], Prabuseenivasan et al. [75], and Brnawi et al. [76] reported antibacterial activity of cinnamon oil against several Gram-positive and Gram-negative bacteria. The antimicrobial activity of cinnamon oil can be ascribed to eugenol, a phenylpropene that was found in this work at 95.23%. This compound alters the membrane and the transport of ions and ATP and modifies the fatty acid profile [63]. Eugenol is active against foodborne pathogens such as *E. coli*, *L*. *monocytogenes*, *S. aureus*, *S. Typhimurium*, *Bacillus subtilis*, and *B. cereus* [69,77,78].

On the contrary to most of the EOs, essential oils obtained from citric fruits exhibited low or no inhibition against all tested bacteria, except the mandarin essential oil that mildly inhibited *S. Typhimurium* with inhibition zones about 14 mm and moderately to *C. albicans* with inhibition zones of 22 mm. D-limonene is one of the major compounds of the citrus essential oils, a monoterpene whose antimicrobial activity depends on the alkyl group [63]. In this work, it was found as a major compound in lemon, mandarin, sweet orange, and pink grapefruit EOs (87.0, 99.0, 99.2, and 99.0, respectively). In addition to limonene, β-pinene, and γ-terpinene, biological precursors of phenolic compounds, were found in lemon at 6.0 y 3.8%, respectively. Some terpenes do not possess high antimicrobial activity when they are used as a single compound. Such is the case of *p*-cymene, one of the most important components of thyme essential oil, which did not show antimicrobial activity against *E. coli*, *S*. *sonnei,* and *S. flexneri* using the agar well diffusion assay [71]. Similarly, 21 terpenoids such as limonene, α-pinene, β-pinene, γ-terpinene δ-3-carene, (+)-sabinene, and α-terpinene showed low inhibition of the bacterial growth, whereas all essential oils exhibited considerable inhibitory effects against 25 different genera of bacteria, including plant pathogens, food poisoning, and spoilage bacteria [79], suggesting that a combination of bioactive compounds in a suitable proportion, is needed to achieve a high and effective overall activity.

The minimal inhibitory concentration (MIC) of essential oils against bacteria and *C. albicans* was determined by the agar dilution method and was expressed in mg/mL (Table 6). MIC values obtained are consistent with previous diameters of inhibition zones. The essential oil obtained from *C. cassia* bark showed the lowest MIC values between 0.14 and 0.28 mg/mL for bacteria, and < 0.14 mg/mL for *C. albicans*. Similarly, cinnamon, thyme and oregano essential oils showed great MIC values for all tested strains showing MIC values between 0.28 and 2.25 mg/mL. A moderate effect was observed with the rosemary, celery, sage, and fennel EOs that showed MIC values between 1.125 and 4.5 mg/mL, and the citric fruits essential oils were the less effective EOs with MIC values from 18 to 72 mg/mL for bacteria, and between 4.5 and 18 mg/mL for *C. albicans*.

### 3.4. Antifungal Activity of Essential Oils against B. cinerea

Antifungal properties of the thirteen essential oils were assessed against *B. cinerea* by agar well-diffusion and agar dilution methods. The percentage of inhibition of mycelial growth and the MIC values were determined after 7 days of incubation (Table 7). Among all the essential oils tested, oregano and thyme EOs showed the highest inhibition (98.5 and 98.2%, respectively), followed by fennel and mint EOs that inhibited 93.8 and 93.1% of the mycelia growth, respectively. Mild inhibition was observed by celery EO (48.8%) and low or no inhibition by EOs obtained from citric fruits (3.4 to 18.4%). Antimicrobial control with ketoconazole inhibited completely the mycelial growth. The minimal inhibitory concentration values ranged from 0.07 to 9 mg/mL. Although thyme and oregano EOs showed the highest mycelial reduction, cassia EO showed the lowest MIC value (0.14 mg/mL) followed by mint, oregano, fennel, thyme, cinnamon, and sage EOs that inhibited the fungus by concentrations of 0.56 to 2.25 mg/mL. Rosemary, celery, orange, lemon, mandarin, and pink grapefruit EOs showed MIC values between 4.5 and 9 mg/mL.

Previous research is in concordance with our results. The cassia and oregano EOs completely inhibited the mycelial growth of *B. cinerea* at 0.5 mg/mL, and concentrations of 250 mg/mL thymol and 300 mg/mL carvacrol inhibited completely its spore germination [80]. Additionally, the growth of *B. cinerea* was completely inhibited by cassia [81], oregano, and rosemary essential oils [82]. Other research reported the antifungal activity of carvacrol, the main compound of oregano EO, and eugenol, the main compound of cinnamon EO, against *B. cinerea* [69]. At 500 ppm, cinnamon EOs completely inhibited the growth of *B. cinerea* from 72 h of contact with the EOs, whereas thyme EO achieved the same from 120 h [83]. According to our results, Palfi et al. [84] reported inhibition of the mycelial growth of *B. cinerea* in the presence of thyme, fennel, peppermint, rosemary, and sage essential oils, whereas lemon oil lacked inhibitory activity.

**Figure 2:**
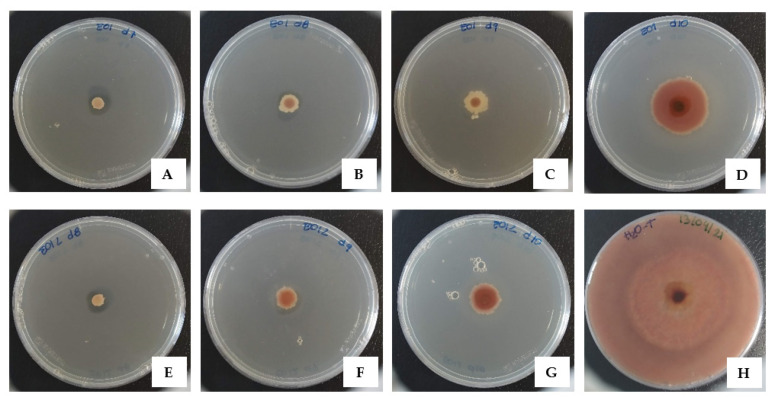
Inhibition of *B. cinerea* mycelium growth by thyme EO at 0.56 mg/mL (**A**), 0.28 mg/mL (**B**), 0.14 mg/mL (**C**), 0.07 mg/mL (**D**); and fennel EO at 0.28 mg/mL (**E**), 0.14 mg/mL (**F**), and 0.07 mg/mL (**G**). Control of mycelium growth (**H**) after 7 days of incubation at 25 °C.

In addition to the dose-dependent effect observed in the MIC assay, some EOs inhibited the fungus by more than 90% at concentrations much lower than their minimum inhibitory concentration. Such is the case of the fennel EO that inhibited 95% of the fungus at a concentration as low as 0.07 mg/mL and thyme EO that inhibited the mycelia growth in 97.6% at 0.14 mg/mL, a concentration 8 times lower than its MIC value (Figure 2). Similarly, only 0.56 mg/mL of the celery EO inhibited 90.2% of mycelia growth, 16 times lower than its MIC value. This result is comparable with those of other researchers, such as the case of Grul’ová et al. [85] that reported complete inhibition of *B. cinerea* with 500 ppm oregano EO, and more than 80% with 100 ppm of oil incorporated into Potato Dextrose Agar.

*B. cinerea* is a phytopathogen that causes the grey mould disease in more than 200 crop species worldwide such as grapes, cucumbers, tomatoes, strawberries, and leading to vast economic losses due to the severe damage in pre-and post-harvest [86,87]. Control strategies are carried out including chemical control, resistance induction and biological control. Benzimidazoles, dicarboximides, phenylpyrroles, aromatic hydrocarbons, and phenylcarbamates are the main chemical fungicides used to control it [88]. In addition to the toxicological risk presented by their residues, *B. cinerea* has developed resistance to most of these substances [89,90,91]. Essential oils have a significant interest as an alternative to chemical treatments as they are bio-sourced products and therefore more ecological [92].

Essential oils can act on fungus via inhibition of sporulation or producing cell damage [93]. Hydrophobic character enables EOs to break through lipids of cell membranes and mitochondria increasing fungal membranes permeability [92]. The changes in the fluidity may leak electrolytes or cellular contents resulting in protein metabolism alteration and calcium ion concentration [93]. Moreover, permeabilization of out and inner mitochondrial membrane leads to cell death by apoptosis and necrosis [94].

Although most of the antimicrobial activities of essential oils have been attributed to their major components, the total antimicrobial effect is the result of the synergism of all their components [95]. Therefore, the antimicrobial activity is not related to a single mechanism of action, as the essential oils have different bioactive compounds, and each one of them has different structural groups in their composition [96].

## 4. Conclusions

EOs from herbs and spices showed the highest antioxidant activity. Essential oils obtained from aromatic plants showed higher antibacterial and antifungal activity than those obtained from citric fruits. The most effective essential oils were those of *C. cassia*, *T. vulgaris*, and *O. vulgare*. Although fennel essential oil reported the lowest antioxidant activity, it showed very good antimicrobial activity against *B. cinerea*, one of the post-harvest pathogen microorganisms in fruits and vegetables, thus it could be considered in active packaging production or food coating. The strong antimicrobial activity of essential oils and the broad spectrum they showed provide evidence that they may be used for prolonging the shelf life of food products, developing functional foods, and for protecting plants and crops.

## Figures and Tables

**Table 1:** List of the EOs used in this work.

Common Name	Scientific Name	Part Used
Aromatic herbs and spices		
True cinnamon	*Cinnamomum zeylanicum* J. Presl	Leaf
Cinnamon	*Cinnamomum cassia* J. Presl	Bark
Oregano	*Origanum vulgare* L.	Leaf
Thyme	*Thymus vulgaris* L.	Flower/leaf
Rosemary	*Rosmarinus officinalis* (L.) Schleid.	Leaf
Peppermint	*Mentha piperita* L.	Leaf
Sage	*Salvia lavandulifolia* Vahl	Leaf
Fruits and vegetables		
Celery	*Apium graveolens* L.	Seed
Fennel	*Foeniculum vulgare* Mill. var. dulce	Not reported
Mandarin	*Citrus reticulata* Blanco	Peel
Sweet orange	*Citrus sinensis* Osbeck	Peel
Lemon	*Citrus limon* L.	Peel
Grapefruit	*Citrus paradisi* Macfad.	Peel

**Table 2:** Composition (expressed as %) of aromatic plant EOs determined by GC-MS.

	Compound	*Cinnamomum* *cassia*	*Cinnamomum* *zeylanicum*	*Mentha* *piperita*	*Origanum* *vulgare*	*Rosmarinus* *officinalis*	*Salvia* *lavandulifolia*	*Thymus* *vulgaris*
1	Tricyclene	n.d.	n.d.	n.d.	n.d.	0.2 ± 0.02	0.2 ± 0.01	0.08 ± 0.00
2	Thujene	n.d.	0.04 ± 0.00	0.04 ± 0.00	0.3 ± 0.01	0.2 ± 0.00	0.2 ± 0.01	0.6 ± 0.03
3	α-pinene	0.05 ± 0.00	0.2 ± 0.03	0.7 ± 0.07	1.0 ± 0.10	11.9 ± 0.90	5.5 ± 0.30	0.7 ± 0.06
4	Camphene	n.d.	0.08 ± 0.00	0.06 ± 0.00	0.2 ± 0.00	5.0 ± 0.30	7.7 ± 0.40	0.7 ± 0.01
5	Sabinene	n.d.	n.d.	0.2 ± 0.00	n.d.	n.d.	1.1 ± 0.30	0.004 ± 0.00
6	β-pinene	0.03 ± 0.00	0.07 ± 0.00	0.9 ± 0.02	0.07 ± 0.01	2.5 ± 0.30	5.1 ± 0.20	0.1 ± 0.01
7	β-myrcene	n.d.	0.008 ± 0.00	n.d.	0.5 ± 0.30	30.7 ± 0.40	2.0 ± 0.08	0.7 ± 0.03
8	α-phellandrene	n.d.	0.2 ± 0.01	0.01 ± 0.00	0.07 ± 0.00	0.7 ± 0.02	0.04 ± 0.00	0.1 ± 0.00
9	α-terpinene	0.02 ± 0.00	0.01 ± 0.00	0.1 ± 0.00	0.5 ± 0.02	0.6 ± 0.02	0.3 ± 0.01	1.2 ± 0.10
10	*p*-cymene	0.05 ± 0.00	0.1 ± 0.01	0.3 ± 0.03	5.3 ± 0.20	1.6 ± 0.10	0.3 ± 0.02	16.5 ± 0.30
11	D-limonene	1.5 ± 0.20	0.09 ± 0.00	2.1 ± 0.20	0.2 ± 0.01	2.9 ± 0.30	4.7 ± 0.40	0.3 ± 0.02
12	1,8-cineole (Eucalyptol)	n.d.	n.d.	4.9 ± 0.30	0.07 ± 0.00	14.8 ± 0.50	38.6 ± 0.80	n.d.
13	cis-ocimene	n.d.	n.d.	n.d.	n.d.	0.1 ± 0.00	0.06 ± 0.00	n.d.
14	γ-terpinene	n.d.	n.d.	0.1 ± 0.03	2.4 ± 0.20	0.7 ± 0.02	0.4 ± 0.01	7.1 ± 0.30
15	Terpinolene	n.d.	0.02 ± 0.00	0.05 ± 0.00	0.03 ± 0.00	0.3 ± 0.03	0.2 ± 0.00	0.05 ± 0.00
16	Linalool	1.8 ± 0.10	0.4 ± 0.01	n.d.	0.7 ± 0.04	0.5 ± 0.06	0.7 ± 0.00	2.5 ± 0.08
17	Pinone	n.d.	n.d.	0.004 ± 0.00	n.d.	n.d.	0.3 ± 0.01	n.d.
18	Camphor	n.d.	n.d.	0.09 ± 0.00	0.07 ± 0.00	20.7 ± 0.30	23.6 ± 0.50	0.6 ± 0.02
19	Menthone	n.d.	n.d.	29.8 ± 0.50	n.d.	n.d.	n.d.	n.d.
20	Menthofuran	0.002 ± 0.00	n.d.	1.9 ± 0.30	n.d.	0.004 ± 0.00	n.d.	n.d.
21	Isomenthone	0.002 ± 0.00	0.002 ± 0.00	4.2 ± 0.20	n.d.	0.01 ± 0.00	n.d.	n.d.
22	Isomenthol	n.d.	n.d.	3.6 ± 0.10	n.d.	n.d.	n.d.	n.d.
23	Borneol	n.d.	0.004 ± 0.00	n.d.	0.3 ± 0.04	0.9 ± 0.10	2.0 ± 0.20	1.3 ± 0.10
24	Menthol	n.d.	n.d.	39.7 ± 0.30	n.d.	n.d.	n.d.	n.d.
25	Terpinen-4-ol	n.d.	0.02 ± 0.00	n.d.	0.3 ± 0.00	0.4 ± 0.01	0.4 ± 0.02	1.0 ± 0.1
26	α-terpineol	n.d.	0.04 ± 0.00	0.2 ± 0.00	n.d.	1.0 ± 0.06	0.4 ± 0.01	0.1 ± 0.01
27	Estragole	n.d.	n.d.	n.d.	n.d.	n.d.	n.d.	0.01 ± 0.00
28	Verbenone	0.006 ± 0.00	n.d.	n.d.	n.d.	0.3 ± 0.00	n.d.	0.01 ± 0.00
29	Methyl thymyl ether	n.d.	n.d.	n.d.	n.d.	n.d.	n.d.	0.1 ± 0.02
30	Pulegone	n.d.	n.d.	0.8 ± 0.10	n.d.	n.d.	n.d.	n.d.
31	Linalyl anthranilate	n.d.	n.d.	n.d.	n.d.	n.d.	2.2 ± 0.30	n.d.
32	Piperitone	n.d.	n.d.	0.5 ± 0.03	n.d.	n.d.	n.d.	n.d.
33	Neomenthol acetate	0.003 ± 0.00	n.d.	0.3 ± 0.01	n.d.	n.d.	n.d.	n.d.
34	Cinnamaldehyde	91.9 ± 1.70	n.d.	n.d.	n.d.	n.d.	n.d.	n.d.
35	Bornyl acetate	n.d.	n.d.	n.d.	n.d.	0.8 ± 0.02	0.8 ± 0.01	n.d.
36	Myrtenyl acetate	n.d.	n.d.	n.d.	n.d.	n.d.	1.1 ± 0.02	n.d.
37	Menthyl acetate	n.d.	n.d.	6.1 ± 0.70	n.d.	n.d.	n.d.	n.d.
38	β-isosafrole	n.d.	0.1 ± 0.00	n.d.	n.d.	n.d.	n.d.	n.d.
39	Thymol	n.d.	n.d.	n.d.	1.6 ± 0.20	n.d.	n.d.	61.2 ± 1.10
40	Carvacrol	n.d.	n.d.	n.d.	84.5 ± 1.60	n.d.	n.d.	2.6 ± 0.5
41	Terpinyl acetate	n.d.	n.d.	n.d.	n.d.	n.d.	0.6 ± 0.02	0.06 ± 0.00
42	Eugenol	4.2 ± 0.30	95.2 ± 1.40	n.d.	n.d.	n.d.	n.d.	n.d.
43	β-caryophyllene	0.5 ± 0.02	1.6 ± 0.20	2.7 ± 0.10	1.6 ± 0.10	2.2 ± 0.20	0.7 ± 0.10	1.9 ± 0.20
44	α-caryophyllene	0.03 ± 0.00	0.3 ± 0.04	0.2 ± 0.00	0.1 ± 0.00	0.7 ± 0.08	0.4 ± 0.03	0.05 ± 0.00
45	β-eudesmene	n.d.	n.d.	0.08 ± 0.00	n.d.	n.d.	n.d.	0.07 ± 0.00
46	Eremophilane	n.d.	n.d.	0.03 ± 0.00	n.d.	n.d.	0.2 ± 0.01	0.01 ± 0.00
47	Bisabolene	n.d.	n.d.	n.d.	0.12	n.d.	n.d.	0.07 ± 0.00
48	Isoeugenol	n.d.	0.4 ± 0.10	0.2 ± 0.00	n.d.	0.3 ± 0.03	0.1 ± 0.00	0.07 ± 0.00
49	2-(2-propenyl)-furan	n.d.	0.007 ± 0.00	n.d.	n.d.	n.d.	n.d.	n.d.
50	Benzyl benzoate	n.d.	1.0 ± 0.30	n.d.	n.d.	n.d.	n.d.	n.d.
	Monoterpenes	3.4	1.3	39.9	11.4	58.2	28.9	30.8
	Oxygenated monoterpenes	n.d.	n.d.	5.0	0.1	35.5	62.2	0.6
	Alcohols	n.d.	0.1	43.6	0.5	2.3	2.8	2.4
	Ethers	n.d.	n.d.	n.d.	n.d.	n.d.	n.d.	0.1
	Esthers	n.d.	1.0	6.4	n.d.	0.8	2.5	0.1
	Sesquiterpene hydrocarbons	0.5	1.8	3.1	1.8	2.9	1.3	2.1
	Aldehydes	91.9	n.d.	n.d.	n.d.	n.d.	n.d.	n.d.
	Phenols	4.2	95.7	0.2	86.1	0.3	0.1	63.9
	Others	n.d.	0.2	1.9	n.d.	n.d.	2.2	n.d.

n.d. = not detected.

**Table 3:** Composition (expressed as %) of fruit and vegetable EOs determined by GC-MS.

	Compound	*Apium* *graveolens*	*Citrus* *limon*	*Citrus* *paradisi*	*Citrus* *reticulata*	*Citrus* *sinensis*	*Foeniculum* *vulgare*
1	Tricyclene	n.d.	n.d.	n.d.	0.003 ± 0.00	n.d.	0.004 ± 0.00
2	Thujene	0.009 ± 0.00	0.1 ± 0.02	n.d.	0.01 ± 0.00	n.d.	n.d.
3	α-pinene	0.3 ± 0.04	0.8 ± 0.05	0.1 ± 0.00	0.2 ± 0.02	0.2 ± 0.03	1.2 ± 0.10
4	Camphene	n.d.	0.03 ± 0.00	n.d.	n.d.	n.d.	0.01 ± 0.00
5	Sabinene	0.06 ± 0.00	0.8 ± 0.10	0.2 ± 0.01	0.2 ± 0.00	0.2 ± 0.00	0.03 ± 0.00
6	β -pinene	2.4 ± 0.20	6.0 ± 0.30	0.08 ± 0.00	0.05 ± 0.00	0.05 ± 0.00	0.02 ± 0.00
7	β-myrcene	0.4 ± 0.02	0.4 ± 0.10	0.3 ± 0.03	0.4 ± 0.02	0.4 ± 0.01	0.01 ± 0.00
8	α-terpinene	n.d.	0.08 ± 0.00	n.d.	n.d.	n.d.	0.003 ± 0.00
9	Cymene	0.06 ± 0.00	0.1 ± 0.02	0.02 ± 0.00	0.03 ± 0.00	0.02 ± 0.00	0.01 ± 0.00
10	D-limonene	71.4 ± 1.50	87.0 ± 1.20	99.0 ± 1.60	99.0 ± 1.10	99.2 ± 1.50	1.2 ± 0.40
11	1,8-cineole (Eucalyptol)	n.d.	n.d.	n.d.	n.d.	n.d.	0.01 ± 0.00
12	γ-terpinene	n.d.	3.8 ± 0.07	0.04 ± 0.00	0.06 ± 0.00	n.d.	0.01 ± 0.00
13	Terpinolene	n.d.	0.1 ± 0.02	n.d.	n.d.	n.d.	0.03 ± 0.00
14	Fenchone	0.003 ± 0.00	n.d.	n.d.	0.002 ± 0.00	n.d.	0.3 ± 0.01
15	Linalool	n.d.	n.d.	n.d.	n.d.	n.d.	0.001 ± 0.00
16	Pinone	0.01 ± 0.00	0.001 ± 0.00	n.d.	0.002 ± 0.00	n.d.	0.0005 ± 0.00
17	Camphor	n.d.	n.d.	n.d.	n.d.	0.005 ± 0.00	0.003 ± 0.00
18	5-undecen-3-yne, (E)-	1.2 ± 0.30	n.d.	n.d.	n.d.	n.d.	n.d.
19	Menthofuran	n.d.	n.d.	0.001 ± 0.00	0.002 ± 0.00	0.002 ± 0.00	0.001 ± 0.00
20	Isomenthone	0.002 ± 0.00	0.008 ± 0.00	0.001 ± 0.00	n.d.	n.d.	n.d.
21	Isomenthol	n.d.	n.d.	0.01 ± 0.00	n.d.	n.d.	0.001 ± 0.00
22	Borneol	n.d.	0.004 ± 0.00	n.d.	n.d.	n.d.	n.d.
23	Menthol	0.01 ± 0.00	n.d.	n.d.	n.d.	n.d.	n.d.
24	Terpinen-4-ol	n.d.	0.04 ± 0.00	n.d.	n.d.	0.005 ± 0.00	0.001 ± 0.00
25	α-terpineol	n.d.	0.05 ± 0.01	0.06 ± 0.01	0.01 ± 0.00	n.d.	0.002 ± 0.00
26	Estragole	0.01 ± 0.00	n.d.	n.d.	n.d.	n.d.	0.3 ± 0.02
27	Verbenone	n.d.	n.d.	n.d.	n.d.	0.003 ± 0.00	0.002 ± 0.00
28	Anethol	n.d.	n.d.	n.d.	n.d.	n.d.	96.8 ± 1.80
29	β-caryophyllene	0.4 ± 0.02	0.1 ± 0.00	0.1 ± 0.01	n.d.	n.d.	n.d.
30	Bergamottin	n.d.	0.2 ± 0.01	n.d.	n.d.	n.d.	n.d.
31	α-caryophyllene	0.04 ± 0.00	n.d.	n.d.	n.d.	n.d.	n.d.
32	β-eudesmene	9.8 ± 0.20	n.d.	n.d.	n.d.	n.d.	n.d.
33	Eremophilane	1.2 ± 0.04	n.d.	n.d.	n.d.	n.d.	n.d.
34	Bisabolene	n.d.	0.2 ± 0.00	n.d.	n.d.	n.d.	n.d.
35	Isoeugenol	0.05 ± 0.00	n.d.	0.03 ± 0.00	n.d.	n.d.	n.d.
36	1-(2,4-Dimethylphenyl)propan-1-one	2.5 ± 0.30	n.d.	n.d.	n.d.	n.d.	n.d.
37	Allyl phenoxyacetate	8.2 ± 0.40	n.d.	n.d.	n.d.	n.d.	n.d.
38	2-(2-propenyl)-furan	1.9 ± 0.20	n.d.	n.d.	n.d.	n.d.	n.d.
	Monoterpenes	74.6	99.4	99.8	100	100	2.6
	Oxygenated monoterpenes	n.d.	n.d.	n.d.	n.d.	n.d.	0.4
	Alcohols	n.d.	0.1	0.1	n.d.	n.d.	n.d.
	Phenylpropanoids	n.d.	n.d.	n.d.	n.d.	n.d.	97.0
	Esthers	8.2	n.d.	n.d.	n.d.	n.d.	n.d.
	Sesquiterpene hydrocarbons	11.5	0.4	0.1	n.d.	n.d.	n.d.
	Phenols	0.1	n.d.	n.d.	n.d.	n.d.	n.d.
	Others	5.6	0.2	n.d.	n.d.	n.d.	n.d.

n.d. = not detected.

**Table 4:** DPPH Free Radical-Scavenging Capacity of different essential oils from plants and fruits.

Essential Oils	IC_50_ DPPH (mg/mL)
Aromatic plants EOs	
*Cinnamomum cassia*	0.05
*Cinnamomum zeylanicum*	0.01
*Mentha piperita*	22.98
*Origanum vulgare*	0.29
*Rosmarinus officinalis*	11.19
*Salvia lavandulifolia*	41.82
*Thymus vulgaris*	0.31
Fruit and vegetables EOs	
*Apium graveolens*	25.89
*Citrus limon*	49.06
*Citrus paradisi*	87.67
*Citrus reticulata*	38.79
*Citrus sinensis*	47.30
*Foeniculum vulgare*	105.32
Ascorbic acid	0.003

**Table 5:** Measure of inhibition zone diameters (mm) for essential oils against bacterial strains.

Essential Oils	*S. aureus*	MRSA	*E. coli*	*S. Typhimurium*	*L. monocytogenes*	*C. albicans*
*Thymus vulgaris*	33.0 ± 1.0	33.0 ± 2.0	38.0 ± 3.6	41.0 ± 1.0	35.3 ± 5.0	58.0 ± 2.6
*Origanum vulgare*	28.7 ± 5.5	30.7 ± 3.8	33.3 ± 4.2	35.7 ± 1.2	31.7 ± 2.9	50.7 ± 1.2
*Rosmarinus officinalis*	10.7 ± 1.2	10.0 ± 0.0	11.0 ± 1.7	12.7 ± 1.2	N	18.7 ± 2.3
*Apium graveolens*	12.0 ± 2.6	11.3 ± 0.6	N	13.0 ± 1.0	11.7 ± 1.5	20.7 ± 1.2
*Salvia lavandulifolia*	10.3 ± 0.6	10.3 ± 0.6	10.7 ± 0.6	13.3 ± 0.6	N	25.0 ± 3.0
*Cinnamomum zeylanicum*	18.3 ± 2.3	18.3 ± 1.2	21.3 ± 1.2	21.7 ± 1.2	14.3 ± 0.6	34.7 ± 0.6
*Cinnamomum cassia*	36.0 ± 3.5	34.7 ± 2.3	29.0 ± 4.6	28.0 ± 1.7	28.0 ± 2.0	55.7 ± 3.5
*Citrus sinensis*	N	N	N	N	N	N
*Citrus reticulata*	10.3 ± 0.6	10.0 ± 0.0	N	14.0 ± 1.0	10.3 ± 0.6	22.0 ± 1.7
*Citrus limon*	N	N	N	N	N	N
*Citrus paradise*	N	N	N	N	N	N
*Foeniculum vulgare*	N	N	N	12.0 ± 2.0	N	12.7 ± 2.5
*Mentha piperita*	11.0 ± 0.0	11.0 ± 1.0	13.3 ± 1.5	22.3 ± 2.5	N	40.3 ± 4.0
AA	15.3 ± 0.6	17.3 ± 0.6	32.3 ± 4.0	30.7 ± 1.2	19.0 ± 1.0	25.7 ± 1.2

Results are presented by mean values from three experiments ± standard deviations. *S. aureus: Staphylococcus aureus*; MRSA: methicillin-resistant *Staphylococcus aureus*; *E. coli: Escherichia coli*; *S. Typhimurium*: *Salmonella enterica* serovar Typhimurium; *L. monocytogenes*: *Listeria monocytogenes*; *C. albicans: Candida albicans*; N: no inhibition zone; AA: antimicrobial agents (ciprofloxacin 2 mg/mL for MRSA, 0.1 mg/mL for *L. monocytogenes*, 0.01 mg/mL for the rest of bacteria, and ketoconazole 0.01 mg/mL for *C. albicans*).

**Table 6:** Minimum Inhibitory Concentration MIC (mg/mL) for essential oils against bacterial strains.

Essential Oils	*S. aureus*	MRSA	*E. coli*	*S. Typhimurium*	*L. monocytogenes*	*C. albicans*
*Thymus vulgaris*	2.25	1.125	1.125	1.125	1.125	0.56
*Origanum vulgare*	1.125	1.125	1.125	0.56	1.125	0.56
*Rosmarinus officinalis*	36	36	18	9	18	4.5
*Apium graveolens*	4.5	4.5	36	9	4.5	1.125
*Salvia lavandulifolia*	9	9	9	4.5	4.5	2.25
*Cinnamomum zeylanicum*	2.25	1.125	1.125	1.125	2.25	0.28
*Cinnamomum cassia*	0.28	0.28	0.28	0.28	0.14	<0.14
*Citrus sinensis*	72	72	36	36	36	18
*Citrus reticulata*	36	36	36	36	18	4.5
*Citrus limon*	72	72	72	36	36	18
*Citrus paradise*	72	72	72	36	72	9
*Foeniculum vulgare*	36	72	36	4.5	36	2.25
*Mentha piperita*	4.5	2.25	2.25	1.125	4.5	1.125

*S. aureus*: *Staphylococcus aureus*; MRSA: methicillin-resistant *Staphylococcus aureus*; *E. coli*: *Escherichia coli*; *S. Typhimurium*: *Salmonella enterica* serovar Typhimurium; *L. monocytogenes*: *Listeria monocytogenes*; and *C. albicans*: *Candida albicans*.

**Table 7:** Percentage of mycelium inhibition of *B. cinerea* and minimal inhibitory concentration (MIC) of essential oils.

Essential Oils	Mycelium Inhibition (%)	MIC (mg/mL)
*Thymus vulgaris*	98.2	1.125
*Origanum vulgare*	98.5	0.56
*Rosmarinus officinalis*	79.0	9
*Apium graveolens*	48.8	9
*Salvia lavandulifolia*	79.8	2.25
*Cinnamomum zeylanicum*	70.4	1.125
*Cinnamomum cassia*	81.5	0.14
*Citrus sinensis*	3.4	9
*Citrus reticulata*	3.6	9
*Citrus limon*	9.7	4.5
*Citrus paradise*	18.4	9
*Foeniculum vulgare*	93.1	1.125
*Mentha piperita*	93.8	0.56

## Supplementary Materials

The following are available online at https://www.mdpi.com/article/10.3390/biology10111091/s1, Figure S1: GS-MS chromatograms of fruit and vegetables, and aromatic herbs and spices essential oils.
