# Supplementary material for: Essential Oils from Fruit and Vegetables, Aromatic Herbs, and Spices: Composition, Antioxidant, and Antimicrobial Activities
Source: Biology (Basel). 2021 Oct 25;10(11):1091. doi: 10.3390/biology10111091 (PMC8615279; doi:10.3390/biology10111091)
Supplement: Supplementary file 1 [file biology-10-01091-s001.zip › biology-1347995-supplementary.pdf]

Supplementary 1

Cinnamomum casia

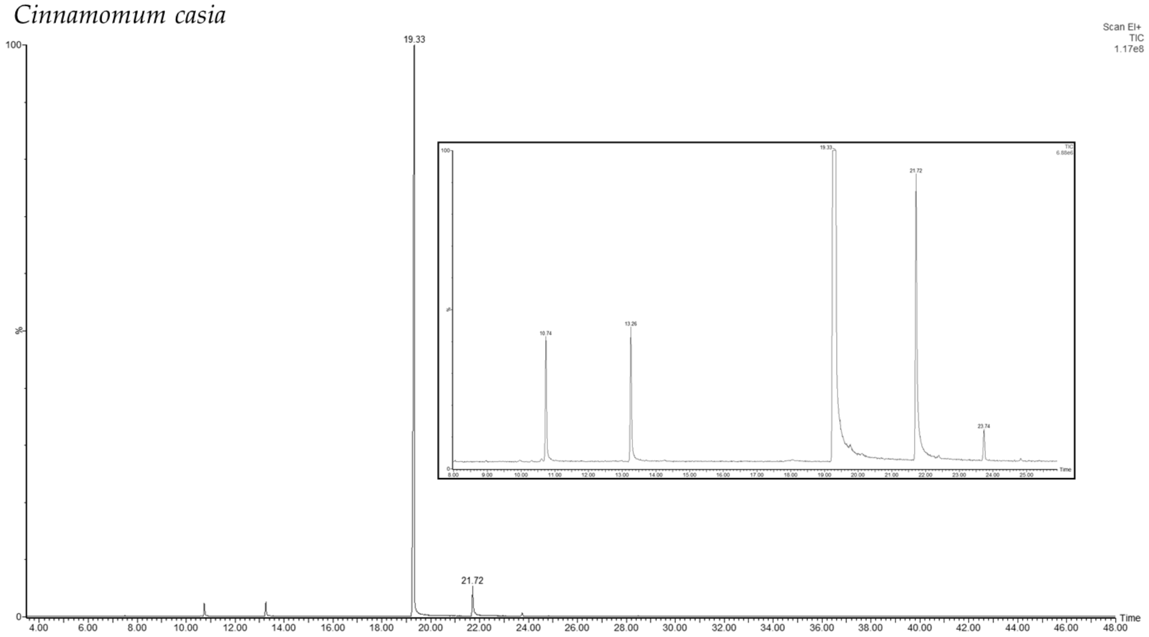

Cinnamomum zeylanicum

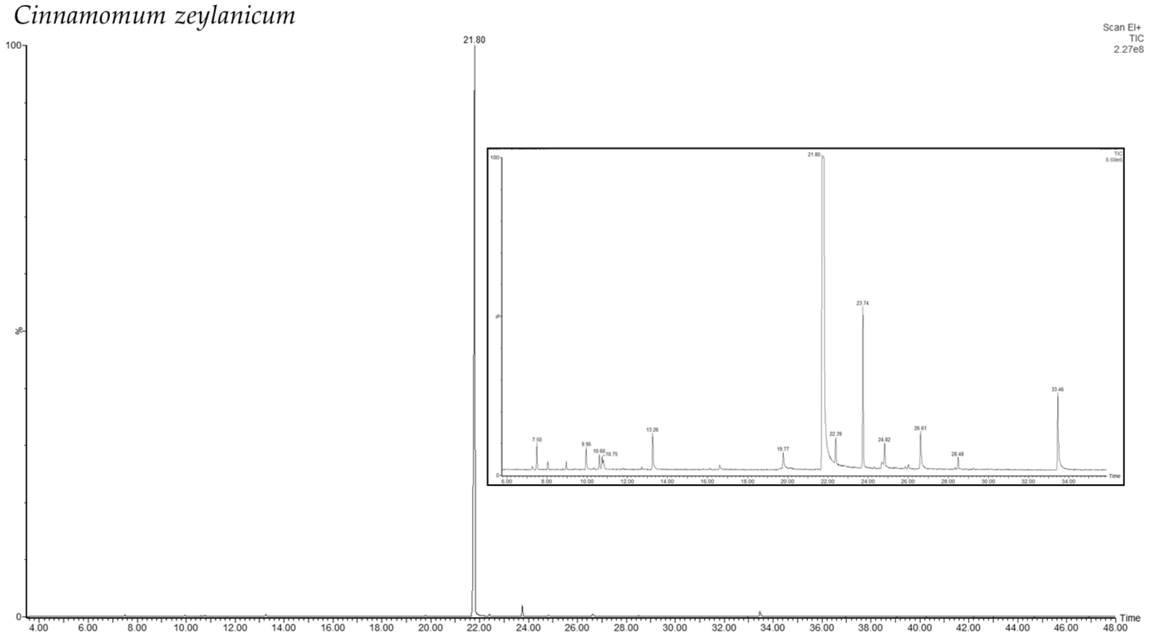

Figure S1: GS-MS chromatograms of fruit and vegetables, and aromatic herbs and spices essential oils.

Mentha piperita

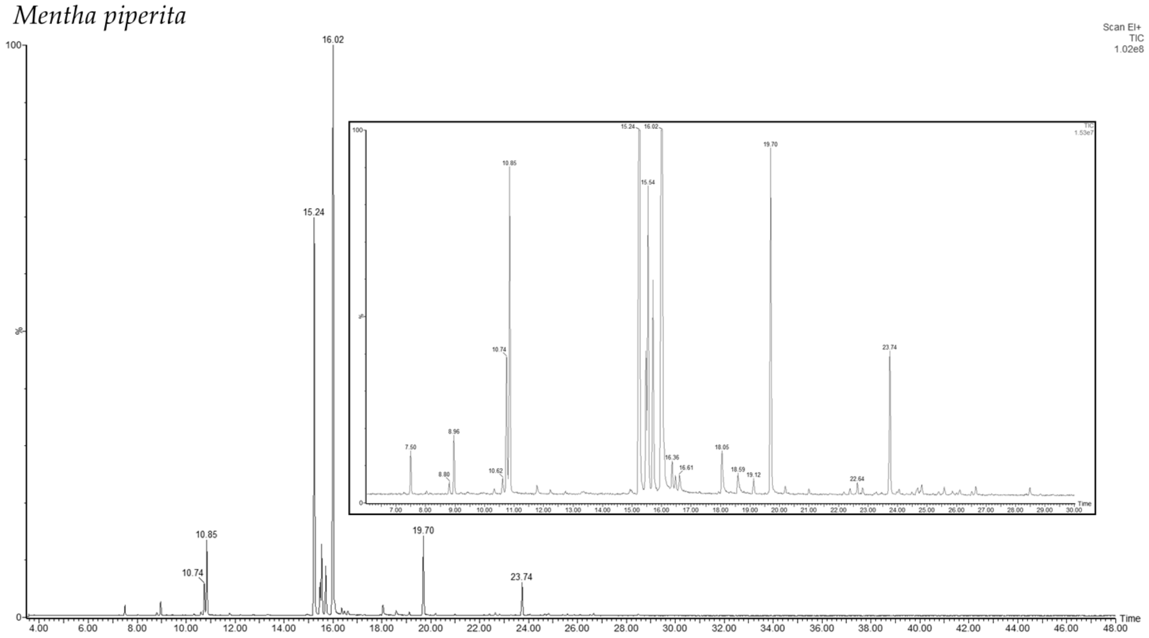

Origanum vulgare

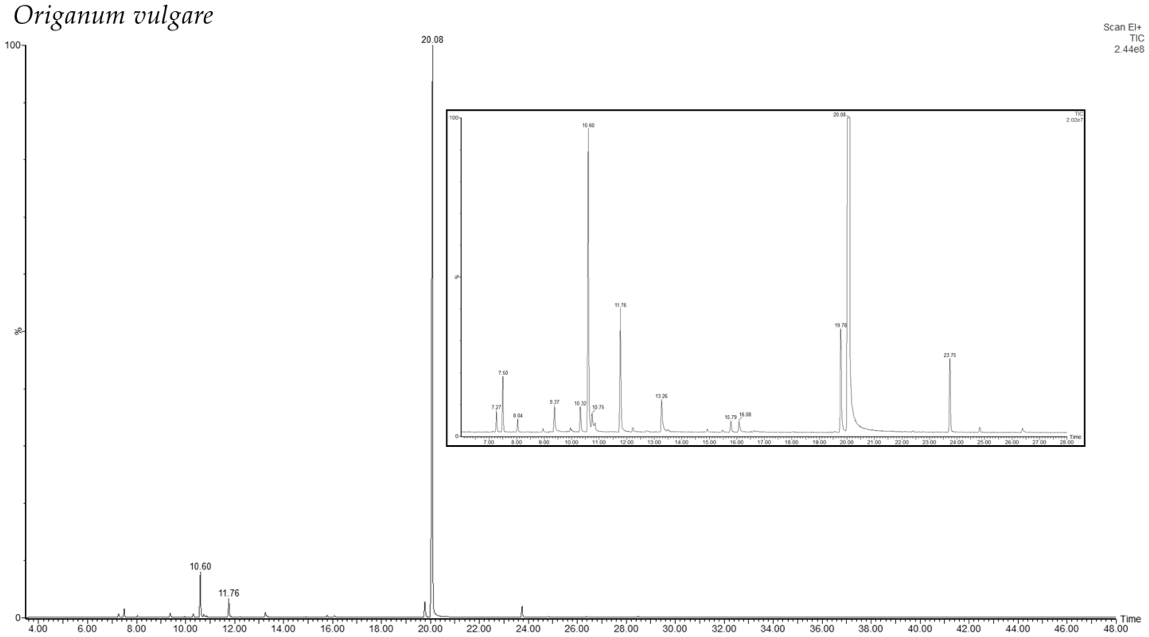

Figure S1 (continuation): GS-MS chromatograms of fruit and vegetables, and aromatic herbs and spices essential oils.

Rosmarinus officinalis

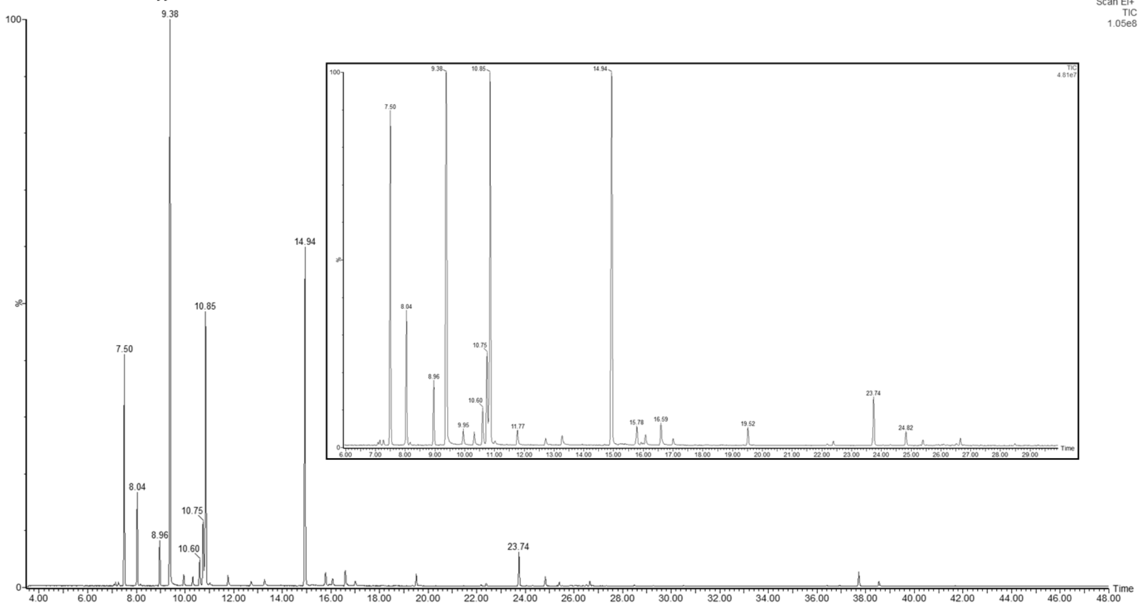

Salvia lavandulifolia

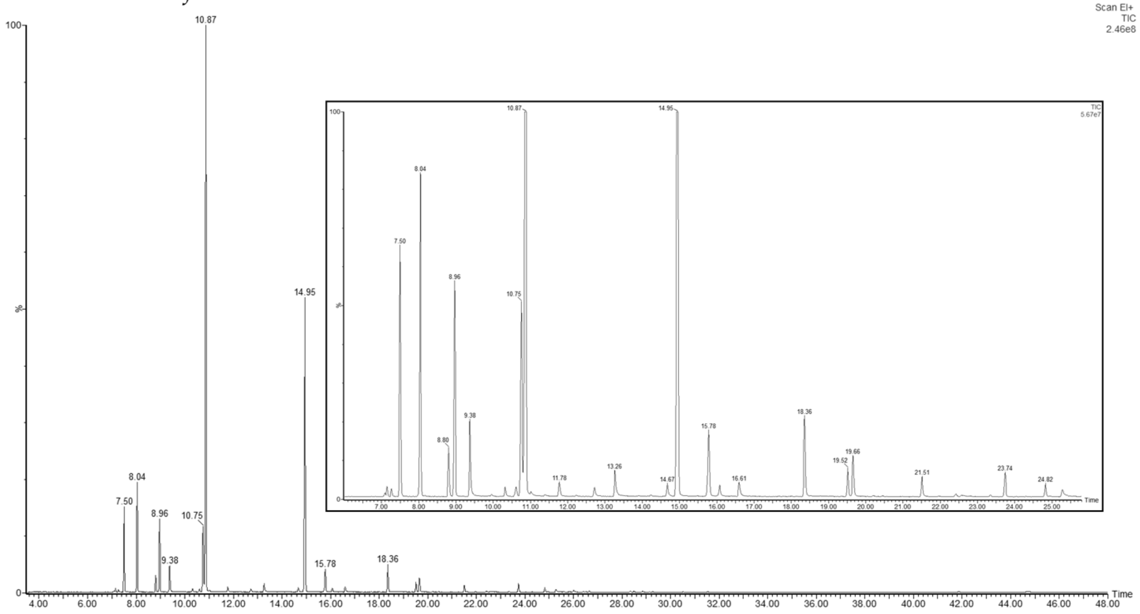

Figure S1 (continuation): GS-MS chromatograms of fruit and vegetables, and aromatic herbs and spices essential oils.

Thymus vulgaris

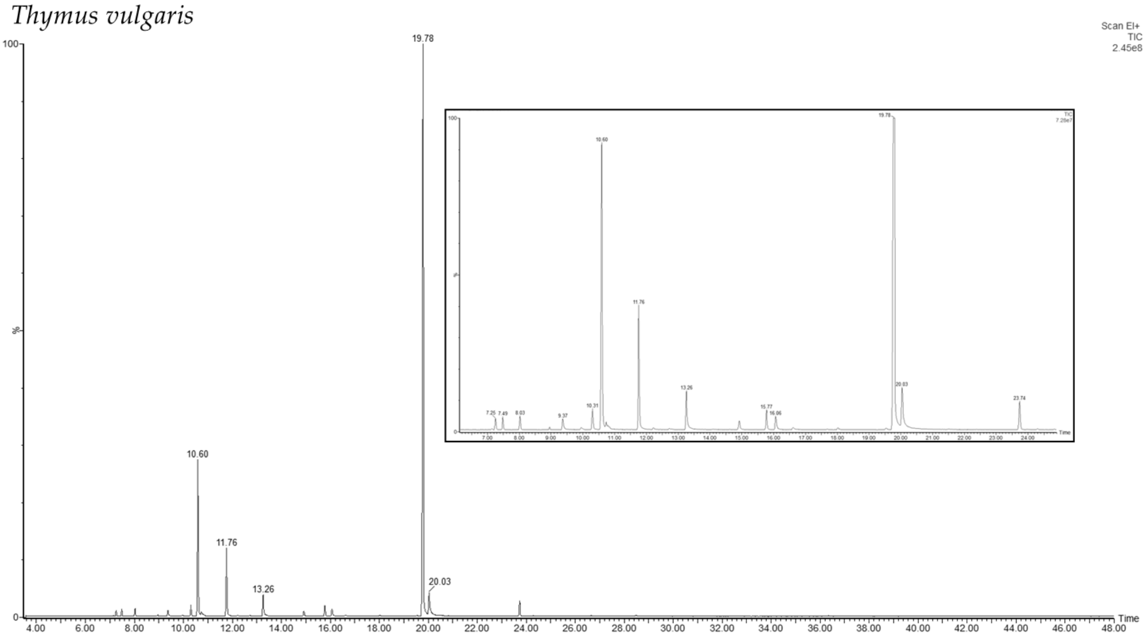

Apium graveolens

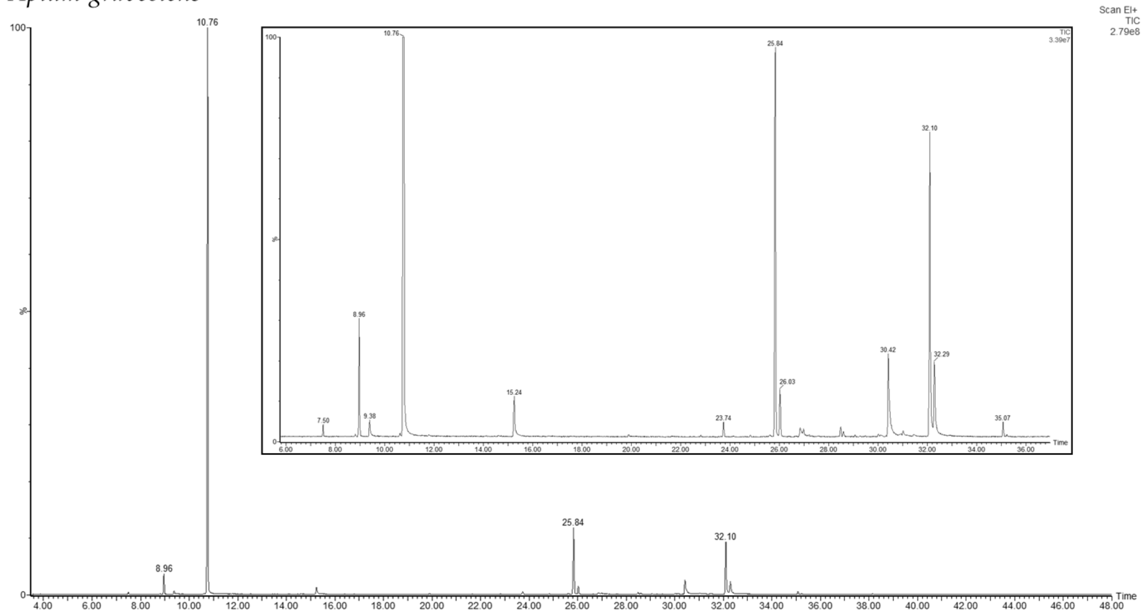

Figure S1 (continuation): GS-MS chromatograms of fruit and vegetables, and aromatic herbs and spices essential oils.

Citrus limon

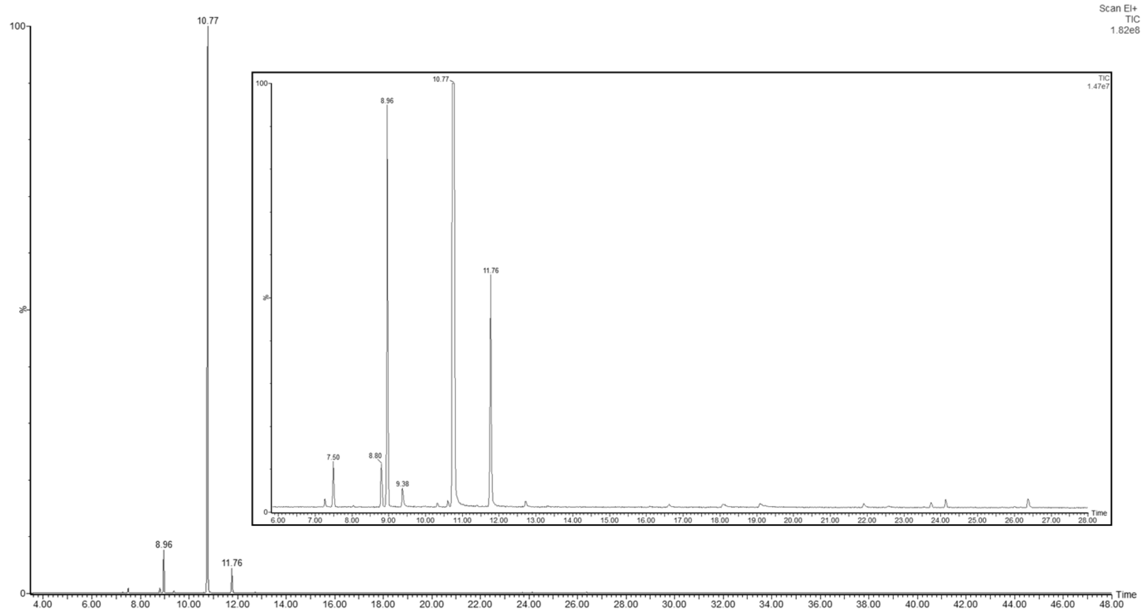

Citrus paradisi

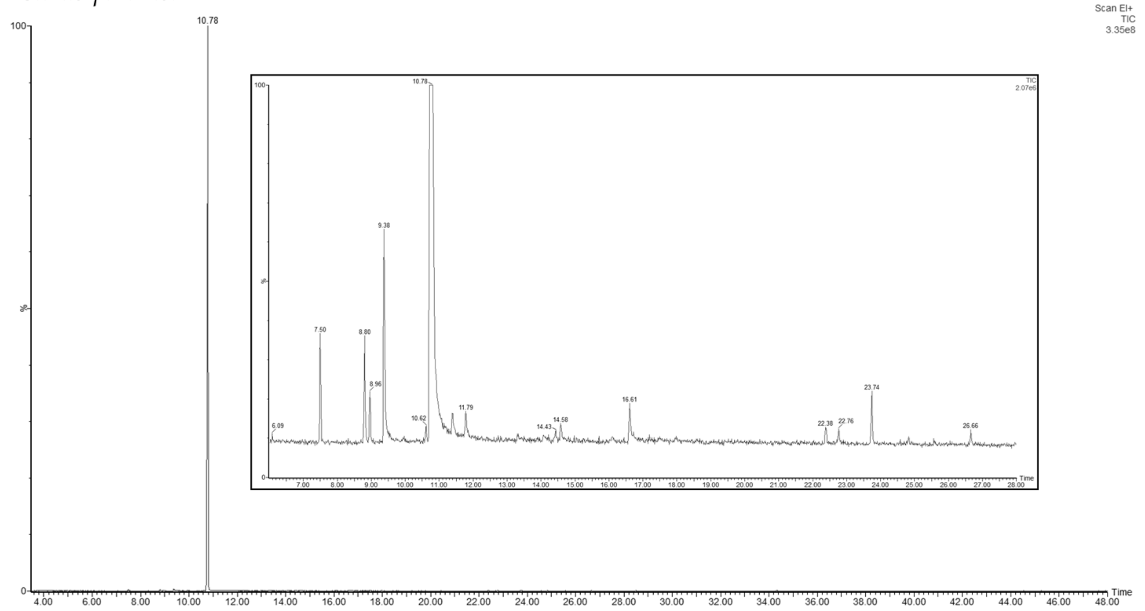

Figure S1 (continuation): GS-MS chromatograms of fruit and vegetables, and aromatic herbs and spices essential oils.

Citrus reticulate

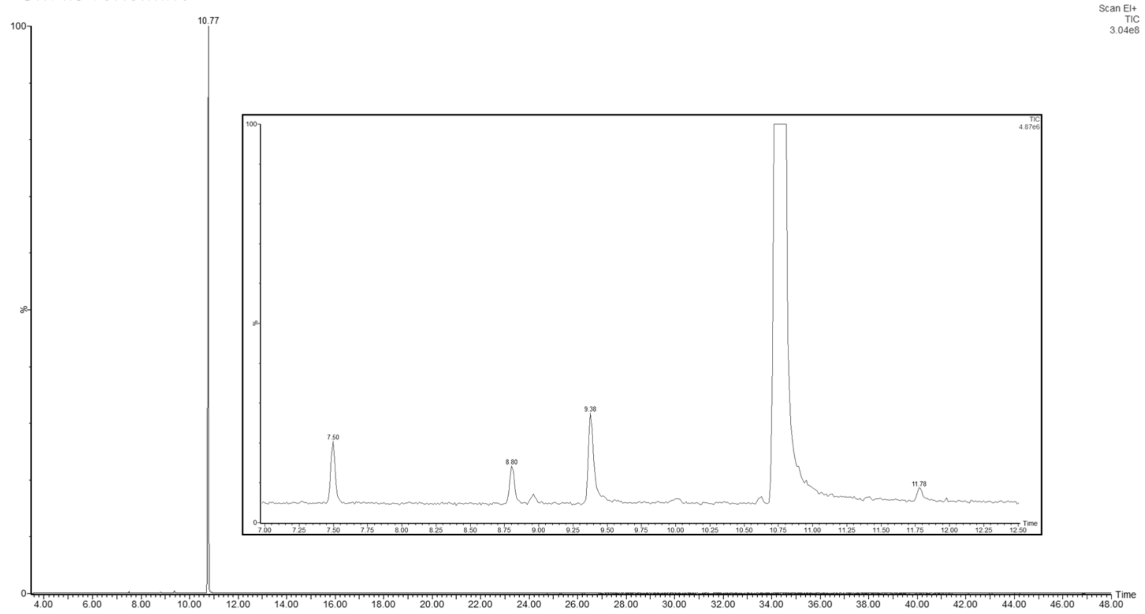

Citrus sinensis

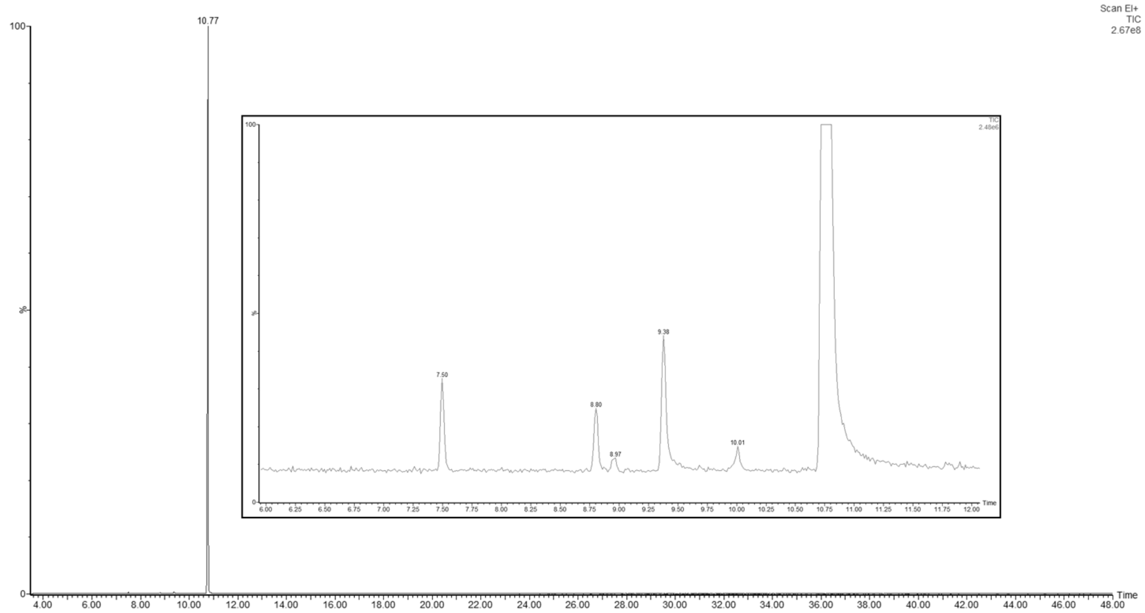

Figure S1 (continuation): GS-MS chromatograms of fruit and vegetables, and aromatic herbs and spices essential oils.

Foeniculum vulgare

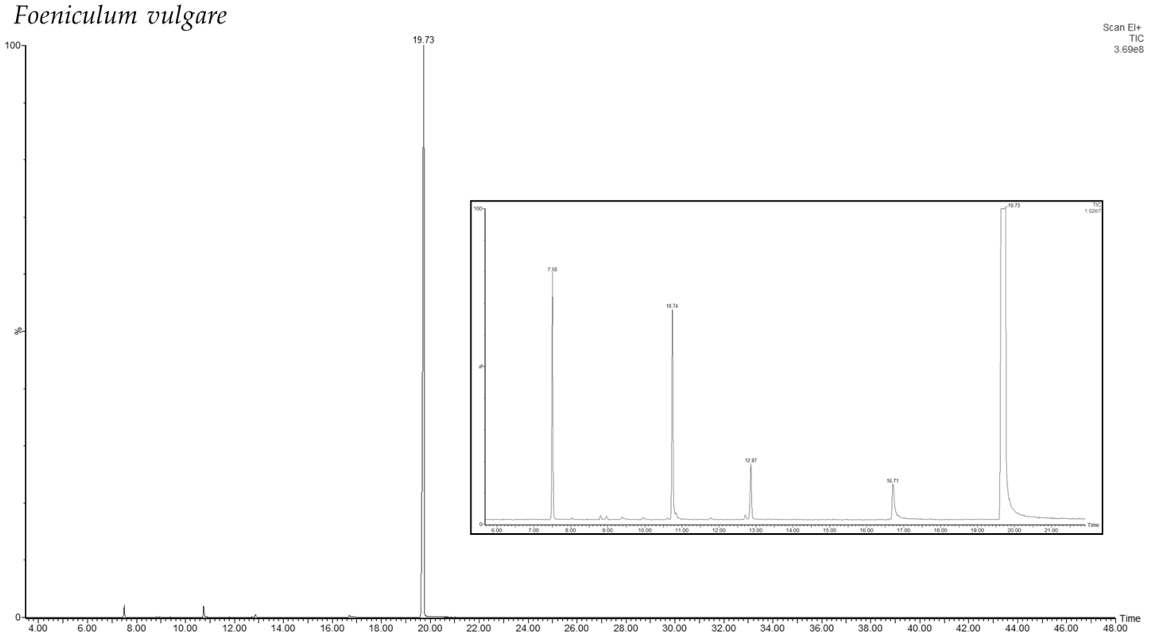

Figure S1 (continuation): GS-MS chromatograms of fruit and vegetables, and aromatic herbs and spices essential oils.
